# Beta-Thalassaemia Intermedia: Evaluation of Endocrine and Bone Complications

**DOI:** 10.1155/2014/174581

**Published:** 2014-07-07

**Authors:** M. Baldini, A. Marcon, R. Cassin, F. M. Ulivieri, D. Spinelli, M. D. Cappellini, G. Graziadei

**Affiliations:** ^1^Rare Diseases Center, Department of Medicine and Medical Specialities, Ca' Granda Foundation IRCCS Ospedale Maggiore Policlinico, 20122 Milan, Italy; ^2^Department of Clinical Sciences and Community Health, University of Milan, Via F. Sforza, 20122 Milan, Italy; ^3^Nuclear Medicine, Bone Metabolic Unit, Ca' Granda Foundation IRCCS Ospedale Maggiore Policlinico, 20122 Milan, Italy

## Abstract

*Objective*. Data about endocrine and bone disease in nontransfusion-dependent thalassaemia (NTDT) is scanty. The aim of our study was to evaluate these complications in *β*-TI adult patients. *Methods*. We studied retrospectively 70 *β*-TI patients with mean followup of 20 years. Data recorded included age, gender, haemoglobin and ferritin levels, biochemical and endocrine tests, liver iron concentration (LIC) from T2*, transfusion regimen, iron chelation, hydroxyurea, splenectomy, and bone mineralization by dual X-ray absorptiometry. *Results*. Thirty-seven (53%) males and 33 (47%) females were studied, with mean age 41 ± 12 years, mean haemoglobin 9.2 ± 1.5 g/dL, median ferritin 537 (range 14–4893), and mean LIC 7.6 ± 6.4 mg Fe/g dw. Thirty-three patients (47%) had been transfused, occasionally (24/33; 73%) or regularly (9/33; 27%); 37/70 (53%) had never been transfused; 34/70 patients had been splenectomized (49%); 39 (56%) were on chelation therapy; and 11 (16%) were on hydroxyurea. Endocrinopathies were found in 15 patients (21%): 10 hypothyroidism, 3 hypogonadism, 2 impaired glucose tolerance (IGT), and one diabetes. Bone disease was observed in 53/70 (76%) patients, osteoporosis in 26/53 (49%), and osteopenia in 27/53 (51%). *Discussion and Conclusions*. Bone disease was found in most patients in our study, while endocrinopathies were highly uncommon, especially hypogonadism. We speculate that low iron burden may protect against endocrinopathy development.

## 1. Introduction

Beta-thalassaemia intermedia (*β*-TI) is a form of NTDT encompassing patients who do not require regular transfusions throughout life, although they may be needed occasionally or even frequently in certain clinical conditions [1, 2]. These patients spontaneously maintain Hb levels 7–10 g/dL and show different levels of splenomegaly and iron overload.

Beta-TI patients show clinical pictures of intermediate severity between the asymptomatic carrier (Thalassaemia Minor) and the transfusion-dependent thalassaemia patients (Thalassaemia Major). The association of mild, moderate, and severe *β*-mutations as well as association of triplication/multiplication of *α*-globin genes can be responsible for wide spectrum of genotypes, and a genotype/phenotype correlation has been observed.

However, several genetic as well as environmental factors can modify clinical expression. The genes affecting the globin chain production are recognized as primary and secondary modifiers, but several other conditions, called tertiary modifiers, can affect the clinical expression, such as the coinheritance of hereditary haemochromatosis or Gilbert syndrome, or alterations of genes involved in iron absorption, bone metabolism, or susceptibility to infections [3]. There is increasing evidence that bone disease may be modified by polymorphisms at loci that are involved in bone metabolism, including the genes for the vitamin D receptor, collagen, and the oestrogen receptor [4–7]. Moreover, varying availability of medical care can influence the outcome of *β*-TI patients.

Endocrine complications are less commonly seen in patients with NTDT than in those with more severe forms of thalassemia (*β*-TM and/or severe forms of haemoglobin E/*β*-thalassaemia) [1, 8–12].

Nonetheless, *β*-TI patients remain at risk of morbidity from endocrine gland and bone pathology, associated with bone pain and fractures [3]. An increased risk of developing several complications, including diabetes mellitus, hypothyroidism, hypoparathyroidism, hypogonadism, and osteoporosis, has been reported in association with higher liver iron concentration (LIC) values or serum ferritin levels as the patients advance in age [13–17]. Conversely, from observational studies lower rates of the same morbidities were associated with iron chelation therapy [11].

As in patients with *β*-TM [18], the pathophysiology of endocrinopathies and low bone mineral density in patients with *β*-TI is probably multifactorial; ineffective erythropoiesis and expansion of the erythron in the bone marrow are directly implicated [1, 19, 20] as well as low foetal haemoglobin, splenectomy, and hydroxyurea therapy [14, 19, 21].

In view of the limited data on endocrinopathy and bone disease prevalence in NTDT, we evaluated these complications in a group of *β*-TI adult patients.

## 2. Patients and Methods

In a retrospective study, 70 *β*-TI adult patients with a mean followup of 20 years were investigated. All patients were followed up at the Hereditary Anaemia Centre of Fondazione IRCCS Ca' Granda Ospedale Maggiore Policlinico of Milan. The study was in adherence to the tenets of the Declaration of Helsinki and approval from the local ethics committee was obtained.

Disease history and systemic treatment, if any, were recorded for all patients including age, gender, haemoglobin and ferritin levels, biochemical and endocrine tests, bone metabolism indices, transfusion regimen, chelation, hydroxyurea therapy, bone specific treatment, and splenectomy.

Patients were considered occasionally transfused when the number of transfusions was <6 per year.

Liver iron concentration (LIC) was derived from T2* from magnetic resonance imaging (Scanner MRI 1,5 Tesla Avanto Siemens, Enlargen - software CMR Tools, Imperial College, London) according to the formula [1/(T2*/1000)] × 0.0254 + 0.202 [21].

All patients underwent bone densitometry scan performed by dual X-ray photon absorptiometry (Hologic Bone Densitometer QDR Discovery A, Version 12.7.3.1 WALTHAM, MA, USA) at the lumbar spine and femur. The bone mineral density values were expressed as *T*- and *Z*-scores; the *T*-score was calculated as a standard deviation score (SDS) from a normal reference population database, while the *Z*-score was calculated as an SDS from an age- and sex-matched population. Data were classified according to the WHO report (WHO Technical Report, ISCD Official Position Paper 2007): *T*-score > −1: normal, *T*-score between −1 and −2.5: low bone density (osteopenia), and *T*-score < −2.5: osteoporosis.

Data are presented as means and standard deviation or median and range as appropriate.

## 3. Results

Among 70 patients, 37 (53%) were males and 33 (47%) were females; mean age was 41 ± 12 years and mean of haemoglobin levels was 9.2 ± 1.5 g/dL. Median ferritin was 537 (range 14–4893) ng/mL and mean LIC 7.6 ± 6.4 mg Fe/g dw. Eleven patients (16%) were anti-HCV antibody positive, and among them 6 (55%) were HCV-RNA positive. Splenectomy had been performed in 34 out of 70 patients (49%) and 13 patients (18%) had a history of bone fractures (2 spontaneous and 11 following traumas).

Regarding patient treatment, 37/70 (53%) had never been transfused and 33 (47%) had been occasionally (24/33; 73%) or regularly (9/33; 27%) transfused. The patients in the latter group were considered NTDT patients because of the regular transfusion regimen that did not start in childhood but starts later, after an occasional event (pregnancy, surgery, infection,…), or because of clinical complications, such as heart failure and fatigue with very low Hb levels leading to poor quality of life. Moreover, the mean transfusional range was 40 days.

Thirty-nine patients (56%) were on chelation therapy and 11 (16%) on hydroxyurea. Hormone defects were corrected with replacement therapy in all cases except one, due to clinical contraindications to sex steroid treatment. Four patients (6%) were on bisphosphonate, 5 (7%) on calcium + vitamin D supplementation, 2 (3%) on calcium alone, and 16 (23%) on vitamin D alone.

Characteristics of the population are summarized in Table 1.

Only 15 out of 70 patients (21%) were diagnosed with endocrinopathy: 10 had hypothyroidism (5 of them were subclinical and did not require therapy), 3 hypogonadism, 2 impaired glucose tolerance (IGT), and one diabetes. Among them, only one patient presented two endocrine complications associated (hypogonadism and diabetes). None of the patients presented with hypoparathyroidism. Vitamin D deficiency was present in 49 patients (70%).

Bone disease was observed in 53 (76%) patients, osteoporosis in 26, and osteopenia in 27 *β*-TI (Table 2).

In the group of patients with osteoporosis, 7 of them (10%) had involvement of both vertebral and femoral sites, 18 (26%) presented only vertebral osteoporosis, and one patient (1%) only had femoral osteoporosis. In the group of patients with osteopenia, 15 (21%) had involvement of both sites; 8 (11%) only had involvement of the vertebral site and 4 (6%) only had involvement of the femoral site. Among the 5 patients with selective involvement of the femoral site (cortical bone disease), BMD was reduced both at the femoral neck and at total femoral scan.

## 4. Discussion

Endocrine data in *β*-TI patients is scanty in the literature. The biggest problem with *β*-TI patients is the wide heterogeneity of the genotypes and of clinical picture, covering a broad spectrum from good general health even in adult age to a compromised condition with early growth retardation and skeletal deformities, due to intense ineffective erythropoiesis and erythroid bone marrow hypertrophy [22, 23]. In such a complex scenario, which also comprises differing genetic patterns and organ damage, the identification of genetic and/or clinical risk factors for endocrinopathy or osteoporosis is a real challenge. Further, bone and endocrine state have been less studied in *β*-TI than in *β*-TM patients.

In our patients with *β*-TI, the prevalence of endocrinopathy does not appear significantly different from that expected in the general nonthalassaemic population [24–26], while bone demineralization, even severe, is a highly common finding, affecting more than two thirds of patients. The prevalence of osteopenia and osteoporosis is very similar, and trabecular bone is predominantly involved regardless of bone disease severity. These findings are in line with previous literature, as bone abnormalities typical of *β*-TM have been described as often present in *β*-TI, and sometimes more marked, starting from the first decade of life. An increased risk of fractures, even spontaneous or following minor traumas, has also been reported; this has been ascribed to osteoporosis as well as to medullary overgrowth due to erythroid stress, aiming to compensate for anaemia [27]. When fractures are recurrent, some authors recommend transfusion therapy [28]. In a large thalassaemic population from North America, Vogiatzi et al. described a 12% prevalence of fractures in *β*-TI patients, increasing with age and with use of sex hormone replacement [29], that is, with hypogonadism.

An additional negative influence on bone condition can be caused by the association between *β*-TI and 25-OH vitamin D deficiency, frequently reported in thalassaemic populations [12, 29]. In our group, vitamin D deficiency was demonstrated in the majority of the *β*-TI patients, 35% of whom were compliant with cholecalciferol supplementation prescribed at the usually recommended dose of 800 IU/day [30]. Nonetheless, bone demineralization of varying severity and low 25OH-vitamin D levels were present in most cases. The persistence of inadequate vitamin D status despite supplementation and the possibility of reduced skin production or impaired liver metabolism of the vitamin have been previously described in *β*-TM patients [31, 32]. If *β*-TI patients had similarly increased vitamin D requirements in order to produce adequate 25-OH D serum levels, they would need high dose supplementation, as indicated by Soliman et al., recommending repletion therapy with 50000 IU of vitamin D weekly for 8 weeks, followed by maintenance with doses up to 50000 IU per month [32].

Unlike *β*-TM, endocrinopathies play a minor role in bone condition in *β*-TI. In a previous study from our group, 78.4% out of 111 *β*-TM patients were hypogonadic, 17.1% were hypoparathyroid, and 16.2% were both hypogonadic and hypoparathyroid, while glucose metabolism was impaired in 40% of patients (diabetes mellitus in 18% and IGT in 22%) [33]. Conversely, in our *β*-TI patients a single endocrine defect was present in merely 15 out of 70 patients (21%), and only one patient (0.01%) had more than one defect.

The low prevalence of endocrine complications in *β*-TI has been previously described; however, in our patients the prevalence of single defects differs from previous reports. More precisely, hypogonadism was exceptional in our group; there were no cases of verified infertility, and more than 50% of our patients had offspring in all cases without induction. In the literature, hypogonadism has been described as the most common endocrine complication in *β*-TI patients, with predominant involvement of females [13, 34]. Moreover, puberty has been described as not infrequently delayed and irregular menses as not unusual, even if generally fertility appeared not to be compromised, with spontaneous conception [35–37]. Diabetes and hypothyroidism appear less common in the literature than hypogonadism; the Optimal Care study found prevalence of 1.7%, 5.7%, and 17% for diabetes, hypothyroidism, and hypogonadism, respectively [13]. By contrast, the thyroid was the most frequently affected gland among our patients (10 cases, 14%), and 24% of them had antithyroid antibodies positivity; this percentage is in line with the high prevalence of autoimmune hypothyroidism in Italy [38]. The prevalence of glucose metabolism impairment in our patients was similar to values described in the literature, while no patient was affected by hypoparathyroidism.

It is well known that the different endocrine involvement between *β*-TI and *β*-TM highlights the different impact of iron overload on the two diseases. In effect, severe iron-overload related endocrine dysfunction is universally described in *β*-TM, while in *β*-TI the pattern of iron overload is preferentially hepatic and it develops gradually throughout life. Compared to the reported data in NTDT [39], our *β*-TI patients showed a very low iron burden, expressed as LIC (mean values 7.6 mg Fe/g dw); this could explain the low prevalence of endocrinopathies, especially regarding the most iron-sensitive cells (gonadotrophs). On the other hand, these LIC values in our series can be ascribed to the nontransfusion-dependent status* per se*, considering that >50% had never been transfused (mean Hb 9.8 g/dL), and all were compliant with iron chelation therapy when prescribed. This is in agreement with the reported association between transfusion therapy (either intermittent or regular) and increased risk of endocrinopathy [13]. In fact the 3 patients with hypogonadism in the present group all had high LIC values and had been transfused.

A further important consideration is the good tissue oxygenation due to the satisfactory Hb levels in our patients, with the mean Hb in the whole series being 9.2 g/dL (evaluating pretransfusion values in regularly or occasionally transfused patients).

Regarding bone mineralization, the group of transfused (both regularly and occasionally) thalassaemic patients showed lower BMD values compared to ones that were not transfused. This finding suggests that when bone damage is established, it persists despite transfusion therapy. In fact, in the group of transfused patients haemoglobin levels had been low for many years before starting transfusional therapy.

We recognize that one limit of our study is the lack of statistical analysis, but this is due to the low prevalence of endocrinopathies, which does not allow any correlation to be drawn. For example, it would be interesting to evaluate if endocrine deficiency can be associated with splenectomy or with hydroxyurea treatment, but this analysis would require a larger population.

For the same reason, we did not divide patients into groups according to transfusion status to avoid an excessive fragmentation of the population.

In view of the scarcity of endocrinological and densitometric data on the *β*-TI population in the literature, unlike the well-studied *β*-TM patients, our findings warrant further studies to evaluate the impact of transfusion and iron-chelation therapy on the outcome of the patients.

## Figures and Tables

**Table 1:** Characteristics of the population.

Parameter	Values
Male (number (%))	37/70 (53)
Female (number (%))	33/70 (47)
Age (years ± SD)	41 ± 12
Hb (g/dL ± SD)	9.2 ± 1.5
Ferritin (ng/mL (range))	537∗ (14–4893)
LIC (mg Fe/g dw ± SD)	7.6 ± 6.4
Anti-HCV positive (number (%))	11/70 (16)
HCV-RNA positive (number (%))	6/11 (55)
Splenectomy (number (%))	34/70 (49)
Transfusion therapy (number (%))	
Never	37/70 (53)
Occasionally	24/70 (34)
Regularly	9/70 (13)
Chelation therapy (number (%))	39/70 (56)
Hydroxyurea therapy (number (%))	11/70 (16)
Hormone replacement therapy (number (%))	2/70 (3)
Levothyroxine therapy (number (%))	5/70 (7)
Bisphosphonate (number (%))	4/70 (6)
Calcium + vitamin D (number (%))	5/70 (7)
Calcium alone (number (%))	2/70 (3)
Vitamin D alone (number (%))	16/70 (23)

*median and range.

**Table 2:** Osteopenia/osteoporosis in 70 *β*-TI adult patients.

Osteoporosis	26/70	Vertebral	18/26		
		Femoral	1/26	Total	0
				Neck	0
				Both	1/1
		Both	7/26	Total	1/7
				Neck	2/7
				Both	4/7

Osteopenia	27/70	Vertebral	8/27		
		Femoral	4/27	Total	0
				Neck	0
				Both	4/4
		Both	15/27	Total	3/15
				Neck	3/15
				Both	9/15

Normal	17/70				
